# The Etiology of Abnormal TSH in Veterans Cared by a VA Medical Center – One High Serum Thyrotropin is Associated with Higher 5-Years Mortality

**Published:** 2023-05-20

**Authors:** Sing-Yung Wu, Mark Chambers, Mazhar Khan, Maureen Chinweze, Thao-My Cao, Haibo Zhao

**Affiliations:** 1 Department of Radiology/Nuclear Medicine, VA Medical Center, Long Beach, California.; 2 Department of Radiological Sciences, University of California, Irvine, California.; 3 Research and Development Service, VA Long Beach Healthcare System, Long Beach, California.

**Keywords:** hyperthyroidism, hypothyroidism, TSH, iodinated contrast, elderly veterans, CV/CNS, CT contrast infusion

## Abstract

**Objective:**

By analyzing the etiology of abnormal TSH in randomly selected veteran patients, we set our heart on improving future clinical care/management of the clinical/subclinical hyper- and hypothyroidism in the aging veteran population.

**Methods:**

A total of 1100 patients’ charts in alphabetical order were selected. Excluded cases of insufficient information, 897 patients’ charts were reviewed and analyzed for causes of abnormal TSH. Among them, 602 for the cause of low TSH (below 0.55 uU/mL) and 295 for high TSH (above 4.78 uU/mL) were reviewed retrospectively.

**Findings:**

Among the 1100 patients selected, 680 (61.8%) were 60 y or older (female=44, 6.8%); 420 were under 60 y (female=80, 19.0%); significantly more female patients were found in the younger age group (P<0.001). After excluding patients with insufficient data, the most common cause of suppressed TSH is iodine-induced, CT iodinated contrast and betadine use caused 35.0% in the older group (n=126) compared to 23.6% in the younger group(n=57) (P = 0.027). The significant difference is that older veterans received more contrast CTs (P < 0.05 compared to the younger group). In both age groups with concurrent FT4 study, we found four high FT4 among 90 studies, 4.4% overt hyperthyroidism. The second most common cause of suppressed TSH is due to thyroid hormone (TH) replacement in the older group (119 patients, 33.1%) with age > 60y, significantly more frequent compared to the younger group, P<0.001. There is significantly more overt hyperthyroidism, 27.8/%, than the iodine-load induced suppression of TSH, P<0.001, due to 17 patients on TSH suppression therapy after total thyroidectomy for thyroid cancer. Among the 295 patients with elevated TSH, the most common cause of high TSH was due to hypothyroidism on T4 replacement: a total of 128 (59.3%) in the older group (N=216) is, similar to 47 (59.5%) in the younger group (N=79). In both age groups, there were 139 patients with concomitant FT4 measurement; 17 overt hypothyroidism were found, 12.2%. No significant difference is seen in the two age groups. The next most common causes of elevated TSH are CT contrast infusion, 23 (10.6%) in the older group and 7 (8.9%) in the younger group. We find high TSH is associated with a higher death rate of 101/238 (42.4%) in a 5-year follow-up (from 2016 to 2021), as compared to low TSH of 68/238 (28.6%), in the older age group, p<0.03； both were significantly higher than the age- and sex-matched general US population, 19.7%, P<0.01.

**Conclusion:**

Even though most, ~ 90%, were subclinical, the suppressed and elevated TSH are associated with severe consequences in CV/CNS and immune-suppression complications in aging veterans. Therefore, cautious use (and more frequent check of TSH) of TH replacement and CT contrast in aging veterans is recommended. The alarming increase in 5 years death rate in older patients with elevated TSH deserves further study.

## Introduction

The veteran population is progressively growing older; the percentage of veterans in age > 60 y was 45.7% in 2000 and increased to 54.9% in 2021 [1]. The thyroid function in the elderly is always an intriguing problem [2]. TSH measurement is a more precise estimation of thyroid function than the studies of thyroid hormones [3]. Abnormal TSH is a commonly encountered finding, of which there are many etiologies. An early British study provided data showing that TSH levels did not vary with age among males but increased markedly among females after the age of 45 years [4]. Data from the National Health and Nutritional Survey (NHANES III) confirmed that both TSH levels and the presence of antithyroid antibodies are greater in women, increase with age, and are more common in whites than in blacks [5].

A low TSH is not always the result of suppression by elevated circulating thyroid hormones, particularly in hospitalized patients. Different etiologies/conditions can yield various levels of TSH. Mildly suppressed TSH without abnormally elevated FT4 is seen in subclinical hyperthyroidism. Markedly suppressed TSH with increased FT4 is found in overt clinical hyperthyroidism. The early recognition of clinical and subclinical hyperthyroidism in the aging veteran population is of increasing clinical importance that may result in severe cardiovascular consequences [6, 7].

On the other hand, subclinical hypothyroidism is not pre-dispose to mental or physical impairment. Strangely, a decrease in thyroid function and a shift to higher TSH values with age may contribute to extended longevity [7, 8]. However, aging VA patients have an alarmingly increased mortality rate with elevated TSH, is shown in the present study.

## Methods

A total of 1100 patients’ charts in alphabetical order were selected. Two hundred-three patients were excluded from the study due to insufficient data, e.g., no VA medication, radiographic study, scanty laboratory tests, or under private physicians’ care outside the VA system. Eight hundred ninety-seven patients’ charts were reviewed and analyzed, among them 602 for the cause of low TSH (below 0.55 uU/mL) and 295 for high TSH (above 4.78 uU/mL) retrospectively.

In the low TSH group, 602 patients’ charts were reviewed and analyzed for the cause of low TSH (below 0.55 uU/mL) retrospectively. In the high TSH category, 295 charts were reviewed. The Chi Square statistic is used for testing relationships between categorical variables [9]. The significant level set at P value < 0.05.

Student’s unpaired t test was used to assess between-group differences. Analysis of variance was used to test multi-group comparisons [9]. Significance was defined as P < 0.05. Results are reported as the mean ± 1 SE. In addition, semi-log linear regression analysis of the serum thyroid functions was used to assess and compare the correlation between TSH and FT4 [10].

## Results

### Causes of Reduced TSH:

In the low TSH group, 770 patients’ charts in alphabetical order were initially examined, and found 441 (57.2%) were 60 years or older (female=30, 6.8%); 329 were under 60 years (female=65, 19.8%). There are significantly more female veterans in the younger age group, P < 0.001.

After excluding patients with insufficient data, 602 patients’ charts were reviewed and analyzed for the cause of low TSH (below 0.55 uU/mL) retrospectively (Table 1). The most common cause of suppressed TSH is iodine-induced; CT iodinated contrast and skin disinfectant use caused 35.0% in the older group compared to 23.6% in the younger group (P=0.027). The significant difference is due to older veterans receiving more contrast CTs (P<0.05 compared to the younger group).

The second most common cause of suppressed TSH is due to thyroid hormone (TH) over-replacement in the older group (119 patients, 33.1%) with age > 60 years, significantly more frequent compared to the younger group, P<0.001(Table 1). The suppressed TSH due to unknown causes is much more frequent in younger patients (p<0.001).

### Cases of Hyperthyroidism after Receiving Iodinated Contrast Agent:

**Figure 1:**
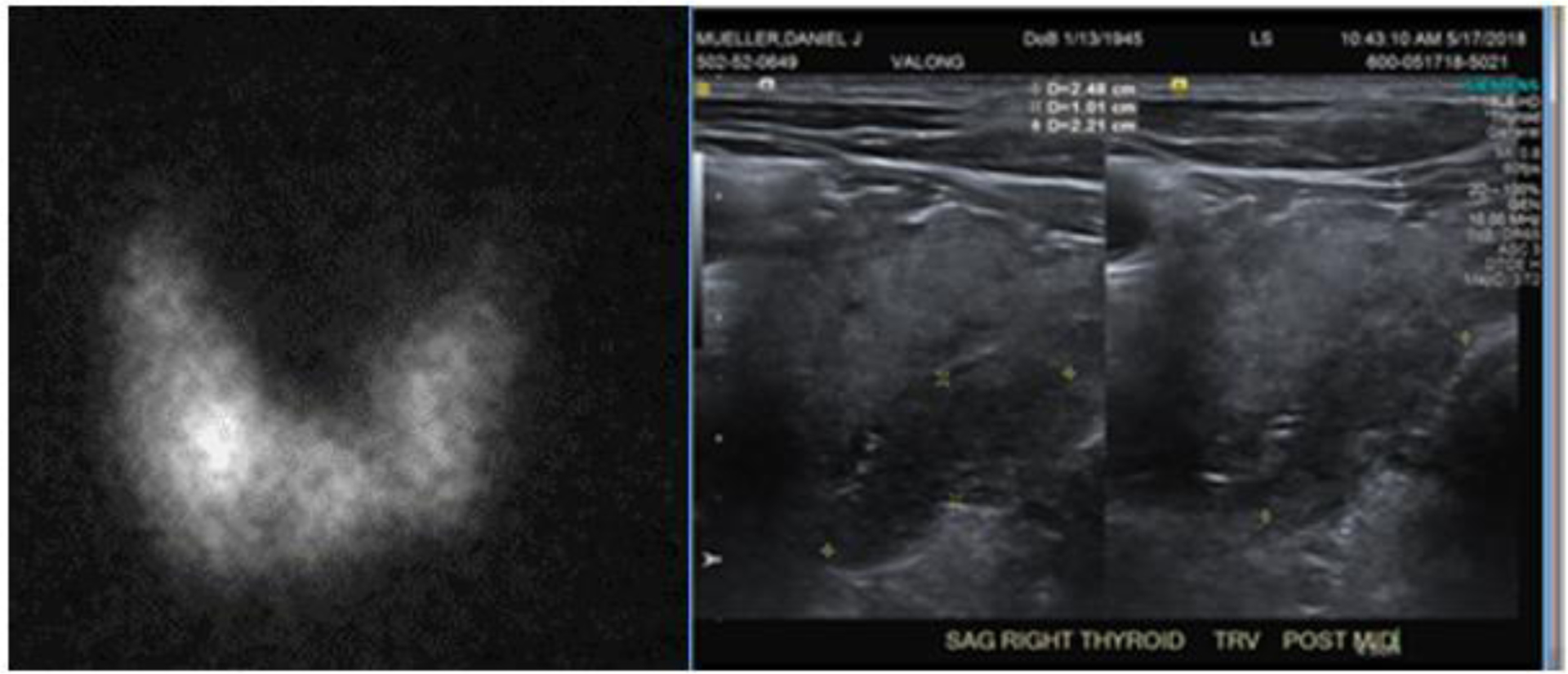
Patient A: (Left) I-123 thyroid scan on 6/27/2018 showed diffusely increased uptake bilaterally (57.5% at 24 h, normal range: 8–35%) and a hot thyroid nodule in the right lower lobe. (Right) Thyroid ultrasound examination on 5/17/2018. The image is the sagittal (long) and trans-axial images There is a 1.8 × 1.3 × 2.1 cm predominantly solid nodule in the medial right lower lobe.

**Figure 2:**
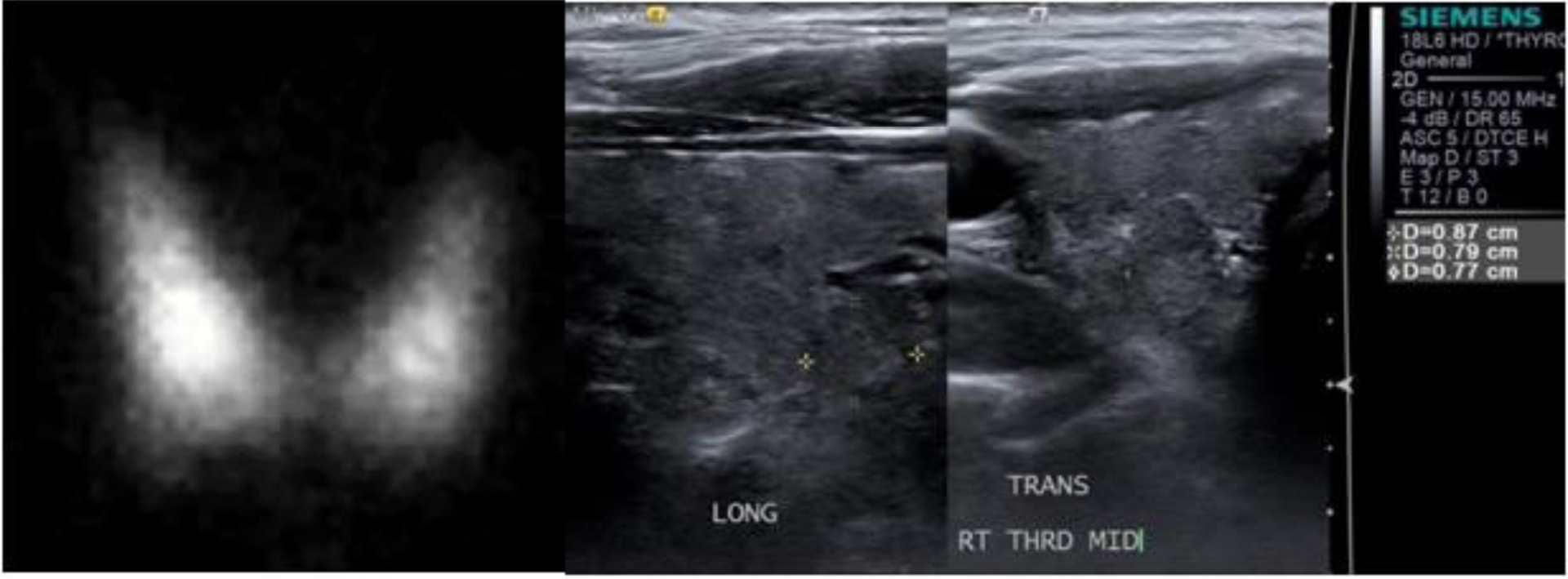
Patient B: (Left) I-123 thyroid scan on 2/26/2015 showed diffusely increased uptake bilaterally (53.9% at 24 h) and a hot thyroid nodule in the right lower lobe. (Right) Thyroid ultrasound examination on 1/13/2017. The image is the sagittal (long) and trans-axial images of the nodule itself with measurements (0.87 × 0.79 × 0.77 cm).

We report six cases of clinical hyperthyroidism, confirmed by thyroid uptake and scan, after receiving an iodinated contrast agent when performing CT scanning (Table 2; Figures 1 and 2).

Patient A. 73 y male, has been euthyroid until after a chest CT angiogram on 3/31/18. He presented to the endocrine service with palpitation and weight loss. Suppressed TSH and elevated free T4 was found on May 18 with a positive thyroid -stimulating immunoglobulin, 458% (reference range <140%). A thyroid ultrasound examination revealed a 1.8×1.3×2.1 cm solid nodule in the medial right lobe (Fig. 1 right panel), where a hot nodule was found on an I-123 thyroid scan with a 24-hour uptake of 57.5% (Fig. 1, left panel). The clinical entity is consistent with the diagnosis of Marine-Lenhard Syndrome induced by an excessive supply of iodine by a CT angiogram [11].

Patient B. A 70-year-old male Vietnam veteran was diagnosed with clinical hyperthyroidism in January 2015, as demonstrated by biochemically suppressed serum TSH and elevated free thyroxine (FT4) concentrations. A history of an iodinated contrast CT performed in April 2013 in a private hospital. Since there was no interim VA medical care, it is presumed that he had some degree of biochemical hyperthyroidism much earlier than that of his presentation following the CT contrast administration. The patient denied palpitations, tremors, and weight loss; there were no thyroid eye disease signs. A thyroid I-123 nuclear uptake and scan in February 2015 showed a single hot nodule in the right lobe with diffusely increased uptake throughout the gland (53.9% at 24 hours; normal 8–35%) (Figure 2, Left). The uptake in the left lobe alone (without any nodule) was 21.6%, markedly elevated. He declined offered treatment with either antithyroid medications or radioactive iodine (RAI) therapy. At a follow-up visit in April 2016, the serum TSH was found to have spontaneously normalized and remained normal in December 2016. An ultrasound examination of the thyroid gland revealed a heterogeneously solid nodule measuring 0.9 cm × 0.8 cm × 0.8 cm in the right lower lobe (Figure 2, right panel) that correlated with the site of the moderately increased uptake on the nuclear scan. Serum thyroid stimulating immunoglobulin (TSI) antibody titers were negative [12]. A repeat thyroid I-123 nuclear uptake and scan showed normal uptake (17.2% at 24 hours) and only a warm nodule in the right lower lobe (Figure not shown).

### Causes of Elevated TSH:

In analyzing the cause, among the 330 patients with elevated TSH, in the older age group (>60 years), the total was 238; 13 were female (5.5%), which was significantly less than females in the younger group, 25.0% (p<0.001). After excluding patients with insufficient data (N=35), the most common cause of elevated TSH was due to hypothyroidism on T4 replacement: a total of 128 (59.3%) in the older group (N=216) is similar to 47 (59.5%) in the younger group (N=79) (Table 3). The next most common causes of elevated TSH are due to iodine load (CT contrast infusion or skin disinfectant), 37 (17.1%) and medications such as amiodarone, lithium, etc., 16 (7.4%) in the older group and 7 (8.9%) and 12 (15.2%) respectively, in the younger group.

Among 295 patients with elevated TSH, 232 had FT4 measurements simultaneously (Table 4). The mean elevated TSH (17.28 mIU/L) in the younger group (N=60) is significantly higher than, the older age group (10.45 mIU/L, N=172, P <0.01) even though the mean FT4 values are the same in the two age groups (Fig. 4).

### Most of the Hypo- and Hyperthyroidism Induced by Iodine Load Or in the Replacement Therapy of Hypothyroidism are Sub-Clinical:

In 183 veterans with iodine-load-induced suppressed TSH, we had 90 patients with FT4 study at the same time in 2016 (Table 5). Only four patients with abnormally high FT4 indicate that about 95.6% of this group is subclinical hyperthyroidism. On the other hand, in the hypothyroid on T4 group, there are 27 having abnormally high FT4; thus, 27.8% are overt hyperthyroidism, significantly higher than the iodine-load group, P<0.001. However, after removing 19 patients with thyroid cancer on T4 suppression therapy, the clinical hyperthyroid cases dropped to 8 cases in 78 (10.2%), no longer statistically significant to the iodine-load-induced hyperthyroidism. In the high TSH group, the subclinical hypothyroidism is similar in Iodineload (92.9%) vs. the On-T4 therapy (87.2%) group (Table 5).

### Thyroid Dysfuncton Induced by Iodine-load:

The suppressed TSH (mean = 0.32) induced by iodine load was significantly lower for the older age group (n=62) as compared with the younger group (mean = 0.38 mIU/L, n=28), P=0.027 (Table 6).

Of 46 veterans with iodine-load-induced elevated TSH, 14 had both TSH and FT4 measurements and found one abnormally low FT4 indicating most are subclinical hypothyroidism. Even though FT4 showed no apparent changes, the mean value (1.14 ng/dL, n=62) in the older age group is significantly higher in the suppressed TSH group than in the elevated TSH group (0.93 ng/dL, n=12), P = 0.003 (Table 6).

### Thyroid Dysfunction found in the T4-therapy Group:

In 602 patients with suppressed TSH, among 157 patients on T4 replacement therapy, 126 had FT4 measurements concurrently. 97 patients were ≥ 60 yrs with significantly higher mean TSH ± SE level (0.22 ± 0.02 mIU/L, normal 0.55–0.78 mIU/L) than the younger group (0.14 ± 0.03 mIU/L, n=29) (Table 7). Twelve thyroid cancer patients on T4 replacement therapy had mean TSH ± SE (0.15 ± 0.04 mIU/L), which is significantly lower than the mean TSH in the older age group (0.22 ± 0.02 mIU/L) (Table 6). Similarly, lower mean TSH is also found in 7 young thyroid cancer survivors on T4 (0.07 mIU/L).

0f patients aged ≥ 80 yrs (N=72, 9 were females), 44 were on TH, reaching 61.1%. Among the 44 patients on T4, 26 had both TSH and FT4 measurements on the same date: mean TSH, 0.20 mIU/L (± 0.03, SE,) and FT4, 1.42 ng/dL (± 0.05, SE, normal 0.67–1.52 ng/dL) (Table 7).

Of 175 patients with elevated TSH and were hypothyroid patients on replacement therapy, 125 had FT4 measurements simultaneously. 92 patients were ≥ 60 yrs with significantly lower mean TSH ± SE level (12.45 ± 1.43 mIU/L) than the younger group (24.92 ± 7.14 mIU/L, n=29) even though the mean FT4 values were similar (Table 7).

### TSH and FT4 Were Found to have Low Correlations:

We plotted the linear correlation in 62 paired TSH and FT4 and found the Pearson correlation coefficient, r = −0.222, poor statistical correlation (Plot not shown). Similar r values are found in TSH and FT4 correlations in the younger age group (n=28) or in two age groups with elevated TSH (Figures not shown).

**Figure 3:**
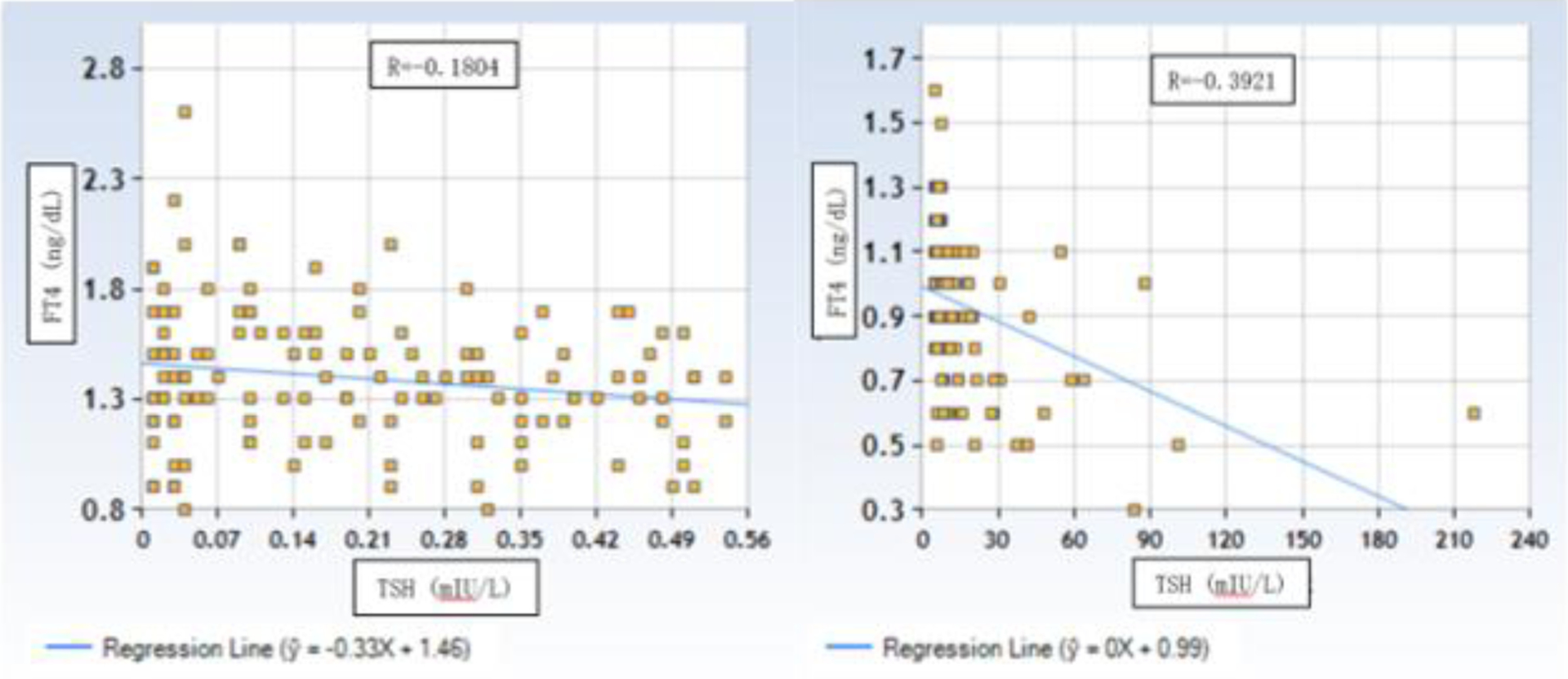
Correlation of TSH and FT4 in 126 veterans with suppressed TSH (left) and 125 veteran with elevated TSH (right) on T4 replacement therapy with hypothyroidism suppressed TSH.

Correlation of TSH and FT4 in 126 veterans with suppressed TSH (Fig. 3, left) and 125 veterans with elevated TSH (Figure. 3, right) on T4 replacement therapy with hypothyroidism suppressed TSH. The Pearson correlation coefficient was R=−0.1804 for the suppressed TSH group and R=−3921 for the elevated TSH group. The FT4 correlates poorly with changing TSH. Similar poor correlations between TSH and FT4 are observed in iodine-load-induced abnormal TSH groups (Figures not shown)

### Other Clinical Aspects in Patients with Elevated TSH:

In the older age group with increased TSH, a total of 44 patients had antibody studies (Table 8); 39 were performed in the male group and found 11were positive for anti-thyroid-peroxidase (TPO) antibody (28.2%), and 7 were positive for anti-thyroglobulin (Tg) antibody (17.9%) (Table 8). However, in all five elderly females tested, both antibodies studied were negative. In the younger age group, 14 males had antibody studies; eight were positive for anti-TPO antibody (4 were positive for anti-Tg antibody), while in the eight female, young veterans, three were positive for Anti-TPO antibodies, and two were positive for the anti-Tg antibody. Interestingly a significantly more positive in Anti-TPO Antibody, but not Anti-Tg antibody, in the younger group than in the older group, P = 0.032. The lack of positive TPO-AB in the elderly group may not contribute to more females in the younger group (36.4% vs. 11.4% in the older group) since all five female patients in the older group had negative TPO antibodies (Table 8).

### Elevated TSH is Associated with a Higher 5-Years Mortality Rate

We find high TSH is associated with a higher death rate of 101/238 (42.4%) in a 5-year follow-up (from 2016 to 2021), in the same numbers of veterans equal or older than 60 yrs of age with low TSH (68/238, 28.6%, p < 0.03) (Table 9). Then we found more veteran in the age groups equal and older than 80 in the high TSH group, 62 vs 44 in the low TSH group, P = 0.11. As compared to age- and sex-matched low TSH, there were 79 deaths in 5 years (33.9%), P=0.162 comparing to the high TSH group (Table 9).

Even though there is no statistical difference between these two aging groups after age and sex-matching, however, the excess 22 deaths in the high TSH group remained to be explained. The 5-year mortality rate is also much higher in the elevated TSH group compared to the age- and sex-matched general US population, 19.7%, P<0.001 [13].

We also find high TSH is associated with a higher death rate in the younger group of 8/92 (8.7%) in a 5-year follow-up, compared to veterans equal or younger than 60 yrs of age with low TSH (3/106, 2.8%, p < 0.05) (Table 10). When we combine these two age groups, the high TSH will have a significantly higher death rate than the alphabetically selected low TSH group (p< 0.006) or age-/sex-matched (p<0.05).

The 5-year mortality rate is also higher in the elevated TSH group compared to the age- and sex-matched general US population, 2.2%, P<0.03 [13]. Due to the number is small, further study is needed.

## Discussion

TSH measurement allows for a more precise estimation of thyroid function than the thyroid hormones studies. However, the diagnosis of overt hyper- or hypothyroidism requires the measurement of FT4 (Figure. 3). In our studies, FT4 is poorly correlated with TSH, which is consistent with prior studies, especially in older patients [14–16].

In veteran patients with suppressed TSH, excluding thyroid cancer patients on T4 therapy, most of these patients are subclinical, ~90% (Table 5); we found a high occurrence of low TSH following an iodine-load(s) in the older age group, which is consistent with older veterans receiving more IV contrast CT studies for cardiovascular (CV) and neoplastic diseases. In the present study, the second most common cause of suppressed TSH is due to thyroid hormone (TH) replacement in the older group (119 patients, 33.1%) with age > 60y; significantly more frequent compared to the younger group, P<0.001. Iatrogenic hyperthyroidism is significantly more common in advanced age. In patients aged> 80 yrs (N=72, 9 were females), 44 were on TH, reaching 56.1%. Among the 44 patients on T4, 26 had both TSH and FT4 measurements showing significantly suppressed TSH (0.20 mIU/L) and close to lower normal limit in mean FT4 (1.42 ng/dL), indicating a significant proportion of this advanced age group with T4 replacement reached overt hyperthyroid range. A critical review and meta-analysis reported a 20% increased mortality in patients with overt hyperthyroidism, and six studies assessing cardiovascular mortality were included [17, 18].

On the other hand, studies have examined the relationship between Subclinical hyperthyroidism and mortality in large populations. A study included 1,191 subjects aged 60 or older at the initial inclusion and thyroid function testing. This 10-year study observed excess mortality from cardiovascular causes in subjects with TSH < 0.5 mIU/L. Further subdivision by mortality causes showed both cardiac disease proper and cerebrovascular disease among the causes [17]. While confirming the association with atrial fibrillation, other studies failed to demonstrate increased mortality in older subjects with subclinical cases [19,20]. Other large meta-analyses showed a slightly increased mortality in subjects older than 65 with the diagnosis of hyperthyroidism [21–24].

In patients with elevated TSH, in the older age group (similar to the younger group), the most common cause of elevated TSH was hypothyroidism on T4 replacement (close to 60%, Table 3). The next most common cause of elevated TSH is CT contrast infusion. Iodine-containing contrast infusion causes the acute suppression of TH synthesis (Wolff-Chaikoff effect) and significantly increases TSH concentrations up to 6.4 mIU/L in about 1/5 of patients within five days [17,25,26].

It is essential to recognize that the elderly are at risk of developing iodine-induced or iatrogenic thyroid dysfunctions, which often are transient and reversible. Thus, the morbidity from excessive therapy can be avoided [12]. In 2019, the number and proportion of CT procedures conducted with no contrast media in the United States exceeded those conducted with contrast media, according to IMV Medical Information Division’s 2019 CT Market Outlook Report [27]. IMV estimates that 52% of the 91.4 million CT procedures conducted in the US in 2019 were performed without contrast media, up from 33% of the 76.0 million procedures conducted in 2007. This indicates that the US radiologists have alerted to the adverse effect of CT contrast media. However, from the ACR Manual on Contrast Media [28], the concern of CT contrast media mainly was on contrast-induced nephropathy, not on thyroid dysfunction in the elderly. The present study may call for attention and further study in contrast-induced thyroid dysfunction. In the 2021 Guideline, the European Thyroid Association (ETA) suggested the measurement of baseline serum TSH in high-risk patients for iodine-containing contrast-induced thyroid dysfunction, especially in the elderly and subjects at risk for cardiovascular diseases. If serum TSH is abnormal, thyroid hormones (T3 and/or T4) should be measured [29]. Prior to CT contrast exposure, ETA suggested a thorough case-finding approach based on identifying those persons most likely to have undiagnosed thyroid disease and measuring thyroid function 3–4 weeks after contrast exposure in high-risk patients for possible hyperthyroidism, especially in the elderly and/or those with an underlying unstable cardiovascular disease [30].

Thyroid peroxidase antibodies can be found in up to 30% of unselected subjects aged 70 and older [4]. This is similar to our result in 28.2% of older male veterans (Table 8). In the NHANES study [5], people > 70 years old with TPO antibodies had an incidence of TSH > 4.5 of about 50%, compared to 10% in the “disease-free” group [30], validating the removal of elderly subjects with thyroid autoantibodies from the “healthy control” groups. In the groups aged 70–79 and older than 80, the 97.5th percentile TSH was found higher (than the normal upper range) at 5.9 and 7.5 mIU/mL, respectively. However, in the same population, total thyroxine decreased with age.

A decreased thyroid function and a shift to higher TSH values with age may contribute to extended longevity [7, 8]. However, we have a surprising but alarming finding in our VA patient populations, a higher TSH group in both young and elderly is associated with a higher mortality rate than the same age groups with suppressed TSH (Tables 9 and 10). We postulate that the higher mortality could be due to compromised immunity in hypothyroidism. A central role of thyroid hormones in the modulation of the immune system is confirmed by the influence of T3 and T4 in cytokine maturation and release. This process involves the activation of MAPKs and is mediated by phosphorylation of the Signal Transducer and Activator of Transcription 1α [31]. Furthermore, hypothyroidism correlates with decreased humoral and immune cell responses [32]. Moreover, levels of circulating thyroid hormones positively match up with immunological reactivity in healthy individuals, such as in the physiological maintenance of lymphocyte subpopulations [33]. Recently, it has been shown that T3 increased the number of IL-17-expressing T lymphocytes by activating dendritic cells in vitro [34]. The association of immunity and higher mortality rate with elevated TSH, although mostly subclinical, deserves further study.

Furthermore, many studies showed that adequate thyroid hormone levels are required to achieve optimal outcome from any surgical procedures [35]. Significant differences are found between patients with subclinical and euthyroid patients undergoing a CV surgical procedures regarding complications [36]. A strong association between non-thyroid illnesses with low T3 at admission and increased risk of post operative myocardial dysfunction and death in patients had coronary artery by-pass grafting [37]. Further clinical trials assessing management in older hypothyroid patients are firmly required [38].

## Conclusion

Because of the vulnerability to CV/CNS and immunosuppression and severe consequences in clinical/subclinical hyperthyroidism and hypothyroidism, as well as higher mortality rate with elevated TSH in the older group, cautious use (and more frequent check of TSH) of TH replacement and CT contrast in aging veterans is recommended. Further, a more extensive control study in the VA population is needed.

## Figures and Tables

**Table 1:** Age comparisons in causes of low TSH in 602 patients’ charts reviewed in a VA medical center

Result Age (yrs)	Total Number	After CT Contrast	After Skin Cleansing	Hypothyroid onT4	Hyperthyroidism	drugs	Causes unknown
≥ 60 (%)	360 (100%)	92 (25.6)	34 (9.4)	119 (33.1)	17 (4.7)	10 (2.8)	88 (24.4)
≤ 59 (%)	242 (100%)	40 (16.5)	17(7.0)	38 (15.7)	17 (7.0)	5 (2.1)	125 (51.6)
P values	≥ 60 vs.≤ 59	0.034	0.336	0.0001	0.402	0.592	0.0001

**Table 2:** Six cases of induced hyperthyroidism after a contrast CT.

Patient (age/sex)	Time after CT-contrast	TSH (.55–.78 mIU/L)	FT4 (.67–1.52, ng/dL)	TSIAb (140% baseline)	RAIU (4h, range: 3–20% 24h, 8–35%)	Thyroid Scan/US (See below)	Treatment
A. 73/M (Ref 11)	1.5 month	0.01	3.8	458%	36.0% (4h) 57.5% (24h)	Patchy ↑uptake with hot nodule	RAI therapy after 2 months treatment of MMI
B.70/M (Ref 12)	<1.6 month	0.01	1.9	93%	24.6% (4h) 53.9% (24h)	Diffuse ↑uptake with hot nodule	No therapy, spontaneous recovered 12 months later
C. 72/M	2 months	0.01	1.9	-	22.1% (4h) 61.6% (24h)	Diffuse ↑uptake; No nodule	MMI therapy
D. 75/M	7 months	0.10	2.0	96%	28.5% (4h) 69.3% (24h)	↑uptake; No nodule	MMI therapy, remission 3 months
E. 56/M	2 months	0.37	1.5	89%	65.4% (4h) 75.7% (24h)	↑uptake; No nodule	MMI therapy
F. 34/M	3 months	0.25	1.2	89%	25.5% (4h) 41.6% (24h)	↑uptake; No nodule	MMI therapy

**Table 3:** Age comparisons in causes of high TSH in 295 patients in a VA medical center.

Result Age (yrs)	Total Number	After CT Contrast	After Skin Cleansing	Hypothyroid onT4 or T3	Hyperthyroidism	drugs	Causes unknown
≥ 60 (%)	216	23 (10.6%)	14 (6.5%)	128 (59.3%)	1	16 (7.4%)	34 (15.7)
≤ 59 (%)	79	7 (8.9%)	2 (2.5%)	47 (59.5%)	1	12 (15.2%)	10 (12.6)
P values	≥ 60 vs. ≤ 59	0.64	0.19	0.92		0.07	0.57

**Table 4:** Thyroid functions in veterans with elevated TSH.

Functions	Total (Mean + SE)	≥ 60 yrs	≤ 59 yrs	P value; ≥ 60 vs. ≤ 59 yrs
Elevated TSH N	232	172	60	
TSH (mIU/L)	12.22 ± 1.28	10.45 ± 0.95	17.28 ± 4.09	<0.01
FT4 (ng/dL)	0.96 ± 0.01	0.96 ± 0.02	0.96 ± 0.03	n.s.

**Table 5:** Thyroid Functions in Veterans with abnormal TSH induced by an iodine-load or replacement therapy in hypothyroidism.

	Low TSH Iodine Loaded	Low TSH On T4	High TSH Iodine Loaded	High TSH On T4
Total Paired study	90	97	14	125
Abnormal FT4	4	27	1	16
% of Overt Hypothyroism or Hyperthyroidism	4.4%	27.8%	7.1%	12.8%

**Table 6:** Thyroid Functions in Iodine-load Induced dysfunction.

Functions	Total (Mean ± SE)	≥ 60 yrs	≤ 59 yrs	t test ≥ 60 vs. ≤ 59
Suppressed TSH (N)	90	62	28	
TSH (mIU/L)	0.34 ± 0.01	0.32 ± 0.02	0.38 ± 0/02	P=0.027
FT4 (ng/dL)	1.15 ± 0.03	1.14 ± 0.02	1.18 ± 0.07	P=0.28
Elevated TSH (N)	14	12	2	-
TSH (mIU/L)	7.79 ± 0.82	7.25 ± 0.75	11.03	-
FT4 (ng/dL)	0.93 ± 0.06	0.93 ± 0.07	0.9	-

**Table 7:** Thyroid functions in hypothyroid patients on replacement therapy with abnormal TSH (with concurrent FT4 measurement).

Functions	Total (Mean±SE)	≥ 60 yrs	≤ 59 yrs	≥ 80 yrs (in group ≥ 60 yrs)	**Thyroid CA Patients on T4 (Age ≥ 60 y)	**Thyroid CA Patients on T4 (Age ≤ 59 y)
Low TSH N	126	97	29	26	12	7
TSH (mIU/L)	0.21 ± 0.01	*0.22 ± 0.02	0.14 ± 0.03	0.20 ± 0.03	0.15 ± 0.04	0.07 ± 0.03
FT4 (ng/dL)	1.40 ± 0.03	1.41 ± 0.03	1.36 ± 0.06	1.42 ± 0.05	1.76 ± 0.07	1.66 ± 0.08
High TSH N	125	92	33	23	-	-
TSH (mIU/L)	15.74 ± 2.20	*12.45 ± 1.43	24.92 ± 7.14	10.59 ± 2.00	-	-
FT4 (ng/dL)	0.94 ± 0.02	0.93 ± 0.02	0.95 ± 0.04	1.01 ± 0.05	-	-

* significantly different from ≤ 59 yrs. Group.

** Thyroid cancer survivors on T4 therapy to suppress TSH.

**Table 8:** Antibody studies in high TSH and older group (>60 years) and compared with younger group (<59).

Age	Sex	Total Number	Anti-TPO	Anti-Thyroglobulin
≥ 60	Male	225	39 (11 Pos, 28 Neg)	39 (7 Pos, 32 Neg)
	Female	13	5 (5 Neg)	5 (5 Neg)
	Total	238	44 (11Pos, 33Neg)	44 (7 Pos, 37 Neg)
≤ 59	Male	69	14 (8 Pos, 6 Neg)	14 (4 Pos, 10 Neg)
	Female	23	8 (3 Pos, 5 Neg))	8 (2 Pos, 6 Neg)
	Total	92	22 (11 Pos, 11 Neg)	22 (6 Pos, 16 Neg)
		Young vs. older (positivity)	P = 0.032	P = 0.377

**Table 9:** Comparison of 5-years mortality rate in low and high TSH group in patients age equal or older than 60 years.

	Number	60–69 yrs	70–79 yrs	80–89yrs	90 yrs+	5 yrs deaths (P value vs. high TSH)
High TSH	238 (13 female) (alphabetical)	96	80	47	15	101
Low TSH	238 (16 female) (Alphabetical)	107	87	30	14	68(p<0.03)
Low TSH	238 (13 female) (Age and Sex-matched)	96	80	47	15	79(p=0.162)
US Public	238 (13 female)	96	80	47	15	47(p<0.001)

**Table 10:** Comparison of 5-years mortality rate in low and high TSH group in patients age equal or younger than 59 years.

	Number	20–29 yrs	30–39 yrs	40–49 yrs	50–59 yrs+	5 yrs deaths (P value vs. high TSH)
High TSH	92 (23 female) (alphabetical)	11	22	19	40	8
Low TSH	106 (24 female) (Alphabetical)	17	30	16	43	3 (p<0.05)
US Public	106 (24 female)	17	30	16	43	2 (p=0.03)
